# Effectiveness of intra-oral botulinum toxin injection in comparison to the extra-oral approach on pain and quality of life in patients with myofascial pain: a randomized clinical trial

**DOI:** 10.1007/s00784-024-06051-0

**Published:** 2024-12-17

**Authors:** Alshaimaa Ahmed Shabaan, Islam Kassem, Inass Aboulmagd, Islam A. Amer, Ahmed Shaaban, Mohamed “ Abd-El-Ghafour”, Shaimaa Mohsen Refahee

**Affiliations:** 1 Department of Oral & Maxillofacial Surgery, Faculty of Dentistry, Fayoum University, Fayoum, Egypt; 2 Consultant Oral and Maxillofacial Surgery, Main University Hospital, Alexandria University, Alexandria, Egypt; 3 Oral & Maxillofacial Radiology Department, Faculty of Dentistry, Fayoum University, Fayoum, Egypt; 4 Maxillofacial, Head and Neck Surgery Unit, General surgery Department, Faculty of Medicine, Sohag University, Sohag, Egypt; 5 Prosthodontic Department, Faculty of Dentistry, Future University, Cairo, Egypt; 6 Department of Orthodontics, Faculty of Dentistry, Cairo University, Cairo, Egypt; 7 Oral and Maxillofacial Surgery Department, Faculty of Dentistry, Fayoum University, Fayoum, Egypt

**Keywords:** Myofascial pain, Trigger point, Botulinum, Ultrasonography-guided injection

## Abstract

**Objective:**

To evaluate and compare the effectiveness of the ultrasound-guided intra-oral and extra-oral transcutaneous injection techniques on the clinical outcome variables in patients with myofascial trigger points within the masseter muscle.

**Materials and methods:**

This prospective randomized trial included 42 patients diagnosed with myofascial pain. Patients were randomly allocated into one of two groups based on the technique of trigger point injection: intraoral and extraoral injection technique groups. Each trigger point was injected with 0.1 ml of botulinum-A toxin guided by ultrasound. Pain intensity, mouth opening, and patient quality of life were monitored six months post-injection.

**Results:**

The pain scores were significantly higher in the extraoral group during all follow-up assessments, whereas the MMO was considerably greater in the intraoral group up to three months of follow-up (*p* < 0.008). However, the difference in MMO ceased to be statistically non-significant after six months of follow-up (*p* = 0.927). Additionally, the patient’s quality of life score was significantly higher in the intraoral group compared to the extraoral group (*p* < 0.001) at both the three- and six-month follow-ups.

**Conclusion:**

The intraoral injection technique might be an effective treatment modality for myofascial trigger points in the masseter muscle. It produces pain relief, increases mouth opening, and enhances the overall quality of life compared to the extraoral injection technique.

**Clinical significance:**

The intraoral injection technique for myofascial trigger points is more effective than the extraoral technique; it reduces the need for additional injections, saves money, and enhances patients’ quality of life.

**Trial registration:**

Clinicaltrials.gov (NCT05673655).

## Introduction

Myofascial pain (MP) is the most prevalent type of non-dental pain in the head and neck region [1]. Studies indicate that myofascial pain affects a significant portion of the population, with estimates suggesting that about 30–85% of individuals may experience it at some point in their lives. Research shows that females are more frequently diagnosed with myofascial pain syndrome than males. Estimates suggest that 40–60% of adult females may experience this condition [2]. It is characterized by the presence of active painful and latent trigger points [3]. These trigger points (TrPs) are presented as hyperirritable spots located within a taut band of skeletal muscle or its fascia. The TrPs are associated with muscle stiffness, weakness, and restricted range of motion [4]. ^,^ [2] Trigger point injections using saline, local anesthesia, botulinum toxin, magnesium sulfate, or platelet-rich plasma are effective in pain reduction and are generally well-tolerated. The needling action of these injections and the substances used contribute to successfully managing of chronic and active trigger points by relaxing the muscle fibers and alleviating pain [5–8]. 

Botulinum toxin A (BTX-A) is one of the clostridium toxins used as a therapeutic agent in the treatment of different medical conditions with muscle spasms, including myofascial pain syndrome [9, 10]. It acts by cleaving a protein called SNAP-25, which is essential for the release of neurotransmitters from the nerve endings. This cleavage prevents the release of acetylcholine, a neurotransmitter that stimulates muscle contraction [11]. Moreover, BTX-A had analgesic effects by inhibiting the peripheral and central release of neurotransmitters like glutamate, calcitonin gene-related peptide, and substance P to sensory areas of the trigeminal ganglia; influencing the pain modulation system by affecting the gamma-aminobutyric acid and opioid systems; decreasing microglia activation; and modulating ion channels [12, 13]. 

However, trigger point (TrP) injection showed non-significant results or recurrence of the myofascial pain after a short time [6]. This is attributed to the masseter muscle anatomy.

Masseter muscle is a complicated masticatory muscle that is composed of 3 parts superficial, middle, and deep coronoid parts. The coronoid part is the deepest, it originates from the inner surface of the zygomatic process of the temporal bone and is inserted into the posterior surface of the upper part of the coronoid process [14]. On the other hand, Akita and Fukino noted that the deep layer of the zygomaticomandibularis muscle is better defined in animals. Buch claimed that the confusion in describing the coronoid part arises because the temporalis and masseter muscles are often fused underneath the zygomatic arch [15, 16].

In addition, the superficial part was separated from the other two parts by a thick, and bulk deep tendinous part that prevented the proper localization of the TrPs in the deep parts of the muscle [17]. 

Subsequently, failure or recurrence of the pain is suspected. In addition, this deep inferior tendon prevents the uneven distribution of the injected material between the different parts of the masseter muscle causing what is called paradoxical bulging of the muscle due to uneven contraction of its different parts [18]. 

Therefore, this study aimed to evaluate and compare the effectiveness of the ultrasound-guided intra-oral and extra-oral transcutaneous injection techniques on the clinical outcome variables in patients with myofascial trigger points within the masseter muscle.

## Materials & methods

Before starting the trial, informed consent was obtained from all 42 patients to participate in the trial and share their clinical images. The study followed the Helsinki principles and the CONSORT criteria [19, 20]. 

### Trial setting, design, and registration (fig: 1)

This study was conducted between September 2022 and December 2023 at the authors’ institutions’ Department of Oral & Maxillofacial Surgery. Clinicaltrials.org (NCT05673655) lists the study’s protocol as approved by the ethics committee of Fayoum University (EC2213).

**Fig. 1:**
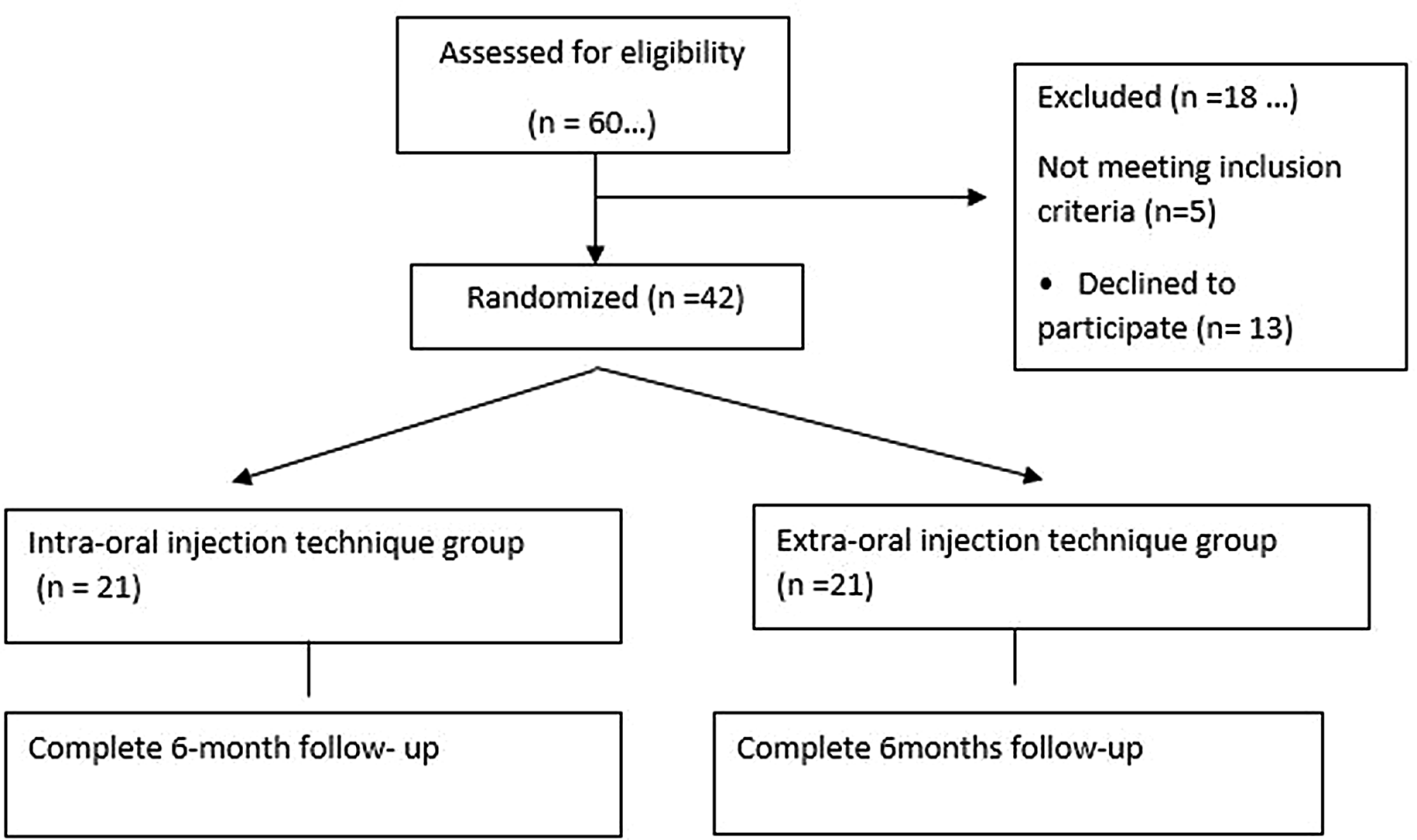
Consort statement flow chart

This prospective, randomized, blinded trial included 42 patients diagnosed with myofascial pain (DC/TMD II.1. A.2) according to the DC/TMD criteria [21]. In addition, they had masseter muscle TrPs on one or both sides with limited mouth opening. All allocated patients had no previous history of orofacial pain other than MP; allergy to Botox, or any systemic condition that could affect the muscles such as fibromyalgia and epilepsy. Those patients who were receiving active treatment for MP or subjected to invasive procedures involving the related masseter muscle were excluded.

### Intervention

Before injection, the operator determines the site of the trigger point by palpation and ultrasound (GE logic Q) with a linear transducer (5–13 MHZ) [22].

Each patient received single-session Onabotulinumtoxin A (BTX-A) (100 U; Botox, Allergan, Irvine, California, CA, USA) injections in the masseter muscles. Botox was dissolved in 2.5 cm sterile saline at room temperature according to manufacturing instructions.

**Fig. 2:**
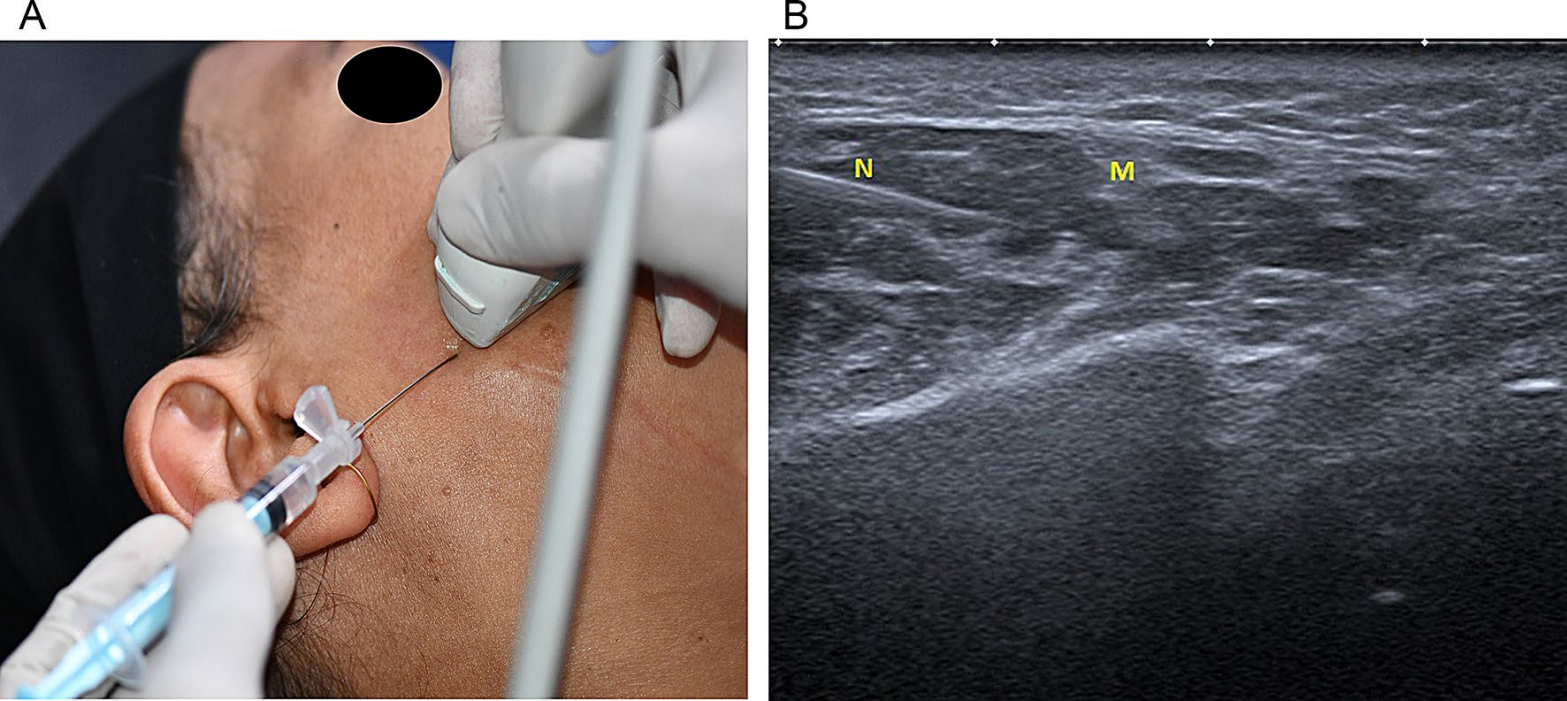
**a**, **b**: Showing the extra-oral injection with guided ultrasound (N: needle; M: masseter muscle)

In ***the extraoral injection technique*** group, the skin was decontaminated with alcohol, and the tight muscle band was detected using ultrasound. A 3/4-inch, 30-gauge needle was advanced with a 30º angle to the skin, and 1–2 cm away from the TrPs to avoid the pain during injection, reduce the risk of complications, and target the surrounding muscle tissue. Negative aspiration was performed and each TrP was injected with 0.1 ml of BTX-A (Fig. 2a, b) [23]. 

**Fig. 3:**
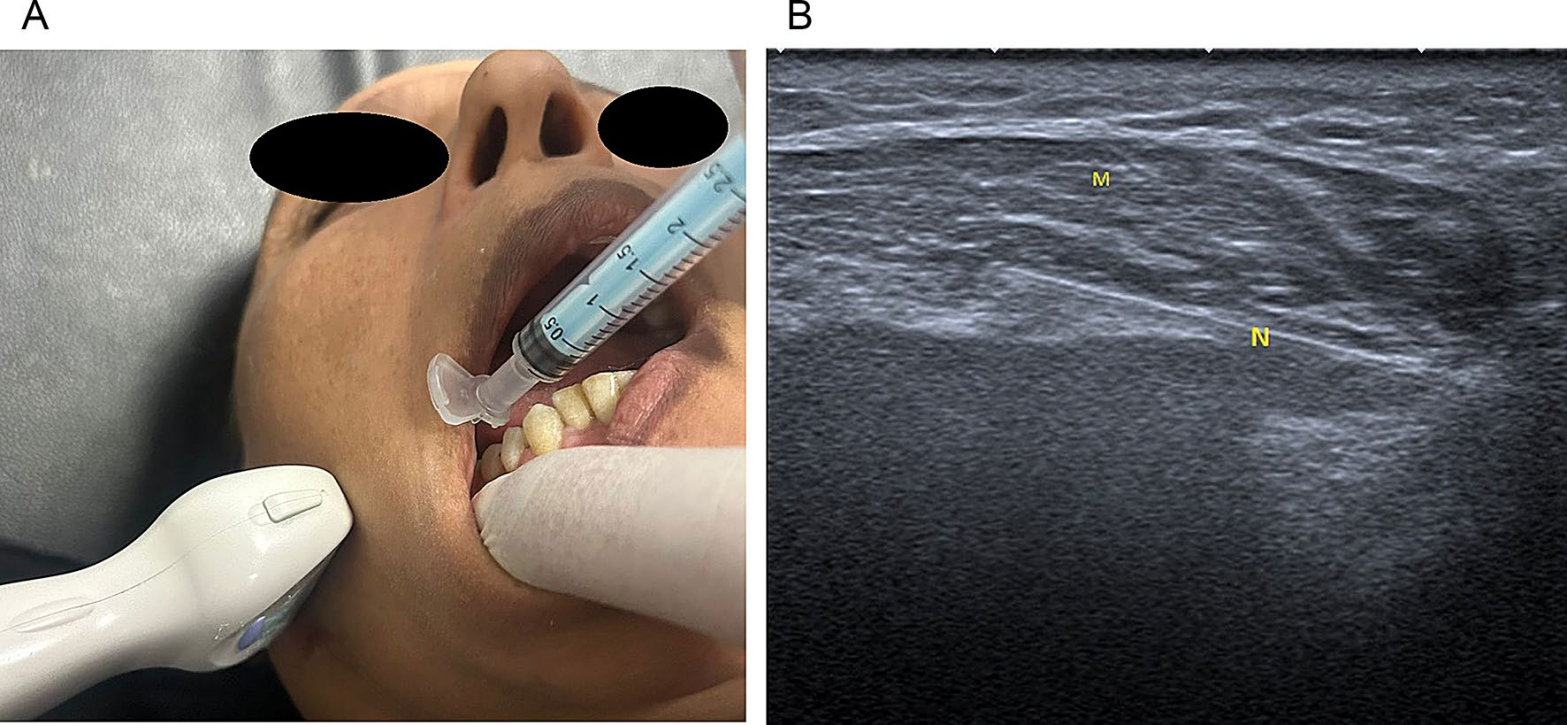
**a**, **b**: Showing the intraoral injection with guided ultrasound (N: needle, M: masseter muscle)

In ***the intraoral injection technique*** group, the patient was asked to clench his teeth, and then the operator held the anterior edge of the masseter muscle between two fingers. The site of injection was disinfected using povidone-iodine. The 30-gauge, 1-inch needle was inserted lateral to the anterior border of the ramus into the muscle as the depth of penetration was guided by the ultrasound to inject each TrP with 0.1 ml of the BTX-A (Fig. 3a, b) [24]. 

### Outcome

All participants were reviewed pre-operatively and then at 1, 3, and 6 months after injection to evaluate the pain intensity, maximum mouth opening (MMO), and quality of life.

The intensity of pain was evaluated by a 10-point visual analog scale (VAS) as (0) implies no pain and (10) implies severe pain [25]. The MMO was evaluated by determining the distance between the cutting edges of the upper and lower central incisors [25]. 

Regarding the quality of life, it was evaluated by the Oral Health Impact Profile questionnaire (OHIP-14) [26]. It includes 14 questions subgrouped into 7 oral health-related domains. The participant was requested to rate all the items on a scale of 1 to 5 [0 = no; 1 = scarcely ever; 2 = occasionally; 3 = pretty often; and 4 = very often]. The total value of all 14 items was subsequently calculated. The OHIP score ranges between 0 and 56 points; a lower value implies no problem, while a higher one suggests a greater quality of life impairment [26]. 

### Sample size calculation

Based on Montes- Carmona et al. [27], the sample size was computed using G*power version 3.1.9.4. The postulated moderate effect size (F = 0.904), power of 0.80, and α = 0.05 were used. Based on this analysis, the study recruited 42 participants divided between 2 groups (*n* = 21 participants per group).

### Randomization and blinding

The randomization was flowing through the 3 steps of random sequence generation, allocation concealment, and implementation. According to the injection technique used, the patients were randomly assigned to two groups using unstratified block randomization with block sizes of 2,4, and 6: Group I (intraoral injection technique) and Group II (extraoral injection technique). Only the study assessors were unaware of the injection technique used. It was not possible to blind both the operators and the patients.

### Statistical analysis

In the current study, the level of significance was set at *p* ≤ 0.05. All the statistical analysis were done with the IBM SPSS (Chicago, Ill.) Statistics version 20 for Windows. Data handling was performed by using Microsoft Excel software.

Data were explored for normality using Kolmogorov-Smirnov and Shapiro-Wilk tests. The means, standard deviations, and confidence intervals were calculated for each group in each test. For each group, a paired sample t-test was used to compare between each two follow-up time points. An Independent sample t-test was used to compare between two groups at the baseline time point, at 1, 3, and 6 months. The same method of comparison was followed for the 3 measured outcomes; VAS scores, MMO, and quality of life questionnaire.

## Results

### Participant baseline data and flow

The current study included 42 participants who were randomly allocated into the two intervention groups (*n* = 21 participants per group). The mean age of the intra-oral injection group was 35.80 years, whereas that of the extra-oral injection group was 33.8 years. Females represented 80.9% (*n* = 34) and males represented 19.04% (*n* = 8) of the total study population (Table 1). There was no dropout in either group during the follow-up periods. Furthermore, no adverse effects or complications related to BTX injections or injection technique. The data of all the included patients was analyzed.

**Table 1:** Participants basic data

Parameters			Intra-oral injection Group *n* = 21		Extra-oral injection Group *n* = 21
Gender	Male n (%)			4 (19.04%)	4 (19.04%)
	Female n(%)			17 (80.9%)	17 (80.9%)
Age	(Mean ± SD)			35. 80 ± 9.53	33.8 ± 8.91

### Outcomes and estimation

The pain scores recorded their highest value at the baseline measurements in both groups and it drops significantly by 1st month post-injection. The pain score recorded a significantly lower value on the intra-oral injection group at all intervals of evaluation post-injection. (Table 2)

**Table 2:** Evaluation of pain score (VAS) of the 2 groups at the different time points and at each time point

Pain (VAS) (follow-up interval)	Intra-oral injection Group *n* = 21 (Mean ± SD)	Extra-oral injection Group *n* = 21 (Mean ± SD)	Difference (Mean ± SD)	*P*- Value
Baseline measurement	7 ± 0.70	7.09 ± 1.09	0.095 ± 0.284	0.738
M1	0.66 ± 0.65	1.28 ± 0.71	0.62 ± 0.212	0.005*
M3	1.28 ± 0.46	2.85 ± 0.35	1.57 ± 0.128	0.008*
M6	1.90 ± 0.83	4.57 ± 0.74	2.67 ± 0.244	0.008*
P-value	0.001*	0.001*		

*; significant (*p* < 0.05), SD; standard deviation; M1: 1 month; M3: 3 months; M6: 6 months follow-up

The MMO showed the lowest value before injection in both groups and improved significantly after injection. Table 3 confirms a significant difference in MMO at 1st and 3rd month post-injection between the two groups while 6th month post-injection showed a non-significant difference between both groups.

**Table 3:** Evaluation of MMO of the 2 groups at the different time points and at each time point

MMO (follow-up interval)	Intra-oral injection Group *n* = 21 (Mean ± SD)	Extra-oral injection Group *n* = 21 (Mean ± SD)	Difference (Mean ± SD)	*P*- Value
Baseline measurement	30.8 ± 1.62	31.68 ± 1.06	0.88 ± 0.044	0.044
M1	35.18 ± 0.88	34.40 ± 0.70	-0.78 ± 0.247	0.002*
M3	35.01 ± 0.94	33.35 ± 0.78	-1.67 ± 0.26	0.004*
M6	32.88 ± 6.82	32.74 ± 0.83	-0.14 ± 1.5	0.927
P-value	0.002*	0.001*		

*; significant (*p* < 0.05), SD; standard deviation.; M1: 1 month; M3: 3 months; M6: 6 months follow up

The OHIP-14 score showed non-significant differences at baseline measurement and 1st month post-injection between the two groups. The scores were significantly lower in the intra-oral injection group compared to the extra-oral injection group throughout the 3rd month and 6th month post-injection (Table 4).

**Table 4:** Evaluation of quality of life using OHIP-14 of the 2 groups at the different time points and at each time point

OHIP-14 (follow-up interval)	Intra-oral injection Group *n* = 21 (Mean ± SD)	Extra-oral injection Group *n* = 21 (Mean ± SD)	Difference (Mean ± SD)	*P*- Value
Baseline measurement	34.66 ± 3.73	34.23 ± 3.25	− 0.43 ± 1.081	0.693
M1	11.71 ± 1.58	13.09 ± 2.75	1.38 ± 0.694	0.053
M3	13.33 ± 1.85	16.19 ± 2.58	2.86 ± 0.693	0.001*
M6	13.76 ± 1.99	23.13 ± 4.17	9.38 ± 1.01	0.007*
P-value	0.001*	0.001*		

*; significant (*p* < 0.05), SD; standard deviation, M1: 1 month; M3: 3 months; M6: 6 months

## Discussion

Botulinum toxin is a Clostridium botulinum neurotoxin. It is commonly used to treat MP due to its ability to relax the muscles and relieve pain [28–30]. It inhibits the presynaptic acetylcholine secretion and pain mediator substances released such as glutamate and substance [11, 12]. The injections were placed away from TPs to avoid pain during injection, reduce the risk of complications, and target the surrounding muscle tissue. Botox can diffuse into the area around the TPs, providing pain relief and muscle relaxation [31]. The Botox can spread during trigger point injections into the surroundings for centimeters depending on the size and the vascularity of the injected muscle as large muscles facilitate wide diffusion. In addition, the botox spread depends on the injection depth as superficial injection allows the wide spread of the toxin. Finally, guided injection with ultrasound helps in the localization of the toxin [17, 32]. Blind botox injection not only allows the wide spread of the toxin, but it may make accidental injection into the parotid gland, facial nerve, or expression muscles causing xerostomia, neuropraxia, or an asymmetric smile [33]. Moreover, missed trigger points can occur with blind injection making the injection insufficient and needing to be repeated more and more causing muscle volume reduction and fibrosis [34]. Consequently, the ultrasound-guided injection maintains the botox within the muscle boundaries and decreases the complications of blindness and repeated injections of botox [34, 35]. 

Although ultrasound-guided injection decreases the complications of botox and increases its efficiency, the deep inferior tendon of the masseter muscle can hinder treatment efficiency by inhibiting the precise accessibility of the needle to the deep part of the muscle [36]. To date, no one has investigated the efficacy of the intraoral injection technique for the masseter muscle TrPs. Thence, the objective of the current study was to evaluate and compare the effectiveness of intraoral and transcutaneous injection techniques on clinical outcome variables in patients with myofascial TrPs within the masseter muscle. The authors suggested that the intra-oral injection technique of the masseter muscle is more accurate, precise, and effective than the transcutaneous one.

In the present study, the VAS scale was used to evaluate the pain intensity at all study intervals. The pain scores recorded their highest value at the baseline measurements in both groups and it dropped significantly by 1st month post-injection. The pain score recorded a significantly lower value in the intra-oral injection group at all intervals of evaluation post-injection, suggesting that this technique may be more effective in managing pain. This improvement could likely be attributed to the analgesic effects of BTX, which impact pain both peripherally and centrally, thereby enhancing the overall effectiveness of the intra-oral technique [28, 29, 30], [37]

Furthermore, the intra-oral injection technique contributes significantly to the reduction of the pain post-injection. This could be likely occurring as the intra-oral injection technique allows the direction of the needle insertion from the lateral side ramus of the mandible would provide better accessibility to the deep and superficial parts of the masseter muscle and provide a more uniform distribution of BTX. Moreover, Procópio et al., [38] and Simons et al., [39] correlated the greater number of nerve penetrations in the deep muscle layer and a larger number of trigger points in the same region.

Besides, the intra-oral injection technique provides better visibility of the TrPs as the transducer goes perpendicular to the injection needle, the beam will hit the needle surface and be reflected again to the transducer. This produces the clearest signal and optimal 2D image quality [40]. 

Regarding the MMO, it showed the lowest value before injection in both groups and improved significantly after injection. A significant difference in MMO at 1st month and 3rd month post-injection between the two groups. Muscle tension, restriction of motor activity, muscle shortening, and spasms can lead to hypomobility. This was in agreement with a study conducted on 20 patients who received 25–50 units of Dysport into their masticatory muscles, mouth opening improved significantly after 4 weeks and persisted for 8 weeks with a concurrent improvement in mandibular movement [41]. Moreover, Sidebottom et al., [42] analyzed the outcome after botulinum injection in patients who did not recover after conservative measures to manage and observed the improvement in the inter-incisal distance was not clinically significant, and concluded that treatment for masticatory myofascial pain does not always improve patients’ capacity to open their mouths and reduction in mouth opening after treatment does not correlate with more or less pain. The significant difference between the two groups supports the better result of intra-oral injection to the muscle as the technique serves to reach more TrPs in the coronoid lobe [40]. 

The pain scores increased, and maximum mouth opening (MMO) decreased at six months in both groups. This change may be due to the fact that BTX works by inhibiting nerve signals to muscles, providing relief from pain and reducing muscle spasms. Over time, the effects of the injection diminish as the nerve endings regenerate and muscle activity returns to baseline levels. Moreover, the muscles may adapt to the initial relief provided by BTX. As the effects wear off, any underlying muscle tension or pain may resurface, leading to increased pain scores and reduced MMO. Furthermore, in chronic conditions, the underlying pathology may continue to progress despite initial treatment, which can contribute to worsening symptoms over time. Additionally, pain perception can be influenced by psychological factors. As the initial relief from pain diminishes, patients may become more aware of discomfort, affecting their reported pain levels [28, 29, 30, 37]

Concerning quality of life, the OHIP-14 score evaluates the comfort improvement and loss of function restoring. The current study’s findings confirm the prior research’s results on the significant improvements in clinical parameters after masseter muscle injection with BTX. In a study using the OHIP-14, Villa et al. [43] found significant improvements in quality of life at 1 and 3 months after injection of BTX to the masticatory muscles of patients with TMD. Another prospective controlled clinical trial investigated the effect of BTX-A on pain control and quality of life in patients with MP and concluded that the pain was reduced, and OHIP-14 improved after 30 days and alleviated myofascial pain [44]. The improvement of OHIP-14 in the intra-oral injection technique significantly over the extra-oral injection technique may be attributed to the accessibility to the deep and superficial parts of the masseter muscle by intra-oral injection technique and it provides a more uniform distribution of BTX.

By evaluating the current study, randomization, prospective patients’ recruitment, and sample size calculation are considered as points of strength. Evaluation of pain, MMO in addition to quality of life is considered as another point of strength. On the other hand, lacking measurements of muscle activity using electromyography to detect any possible alteration in muscle function following the use of intra-oral techniques is considered a limitation. The inability to blind both the operator and patient is another unavoidable limitation of the current study. The lack of a placebo control group could be another limitation lies in the potential for confounding variables to influence the study outcomes. Although the intraoral injection technique has superior results, many limitations could be addressed in future studies, such as the fact that this technique cannot be performed in patients with severe limitations in MMO. This restriction can hinder access to the injection site and may preclude effective treatment for myofascial pain in the affected muscles. Future investigation is needed in addressing these limitations and further investigation regarding the dentist’s perception of the technique and the impact of this technique on complications associated with a repeated injection to the masseter. Moreover, further study could be directed toward combined treatment strategies such as additional physiotherapy, which typically focuses on strengthening muscles and addressing kinesiophobia.

## Conclusion

In conclusion, the findings of the current study suggest that the intra-oral injection technique might be an effective treatment modality for myofascial TrPs of the masseter muscle. It produces pain relief, more maximal mouth opening, and increases the overall quality of life in comparison to the extra-oral injection technique.

## Data Availability

The corresponding author keeps all the study's data

## Abbreviations

BTX-A: Botulinum toxin- A
MMO: Maximum mouth opening
MP: Myofascial pain
OHIP-14: Oral Health Impact Profile Questionnaire
TMD: Temporomandibular disorders
TrP: Trigger point
TrPs: Trigger points
VAS: Visual analog scale
